# The Clinical Aspect of Adaptor Molecules in T Cell Signaling: Lessons Learnt From Inborn Errors of Immunity

**DOI:** 10.3389/fimmu.2021.701704

**Published:** 2021-08-12

**Authors:** Yael Dinur-Schejter, Irina Zaidman, Hagar Mor-Shaked, Polina Stepensky

**Affiliations:** ^1^Faculty of Medicine, Hebrew University of Jerusalem, Jerusalem, Israel; ^2^The Bone Marrow Transplantation and Cancer Immunotherapy Department, Hadassah Ein Kerem Medical Center, Jerusalem, Israel; ^3^Allergy and Clinical Immunology Unit, Hadassah Ein-Kerem Medical Center, Jerusalem, Israel; ^4^Monique and Jacques Roboh Department of Genetic Research, Hadassah Ein Kerem Medical Center, Jerusalem, Israel

**Keywords:** adaptor molecules, ADAP, LAT, SLP-76, T-cell signaling, primary immune deficiency

## Abstract

Adaptor molecules lack enzymatic and transcriptional activities. Instead, they exert their function by linking multiple proteins into intricate complexes, allowing for transmitting and fine-tuning of signals. Many adaptor molecules play a crucial role in T-cell signaling, following engagement of the T-cell receptor (TCR). In this review, we focus on Linker of Activation of T cells (LAT) and SH2 domain-containing leukocyte protein of 76 KDa (SLP-76). Monogenic defects in these adaptor proteins, with known roles in T-cell signaling, have been described as the cause of human inborn errors of immunity (IEI). We describe the current knowledge based on defects in cell lines, murine models and human patients. Germline mutations in Adhesion and degranulation adaptor protein (ADAP), have not resulted in a T-cell defect.

## Introduction

Engagement of the T cell receptor (TCR) triggers a signaling cascade responsible for T-cell activation, maturation and differentiation. Fine tuning of this complex multi-protein cascade enables discriminating different signals based on strength and duration. In the thymus, this process allows for positive selection. In the periphery, weak, self-peptide-MHC survival signals are differentiated from strong foreign-peptide-MHC activating signals (1). Moreover, signaling strength is crucial for determining T-cell fate (2).

**Figure 1:**
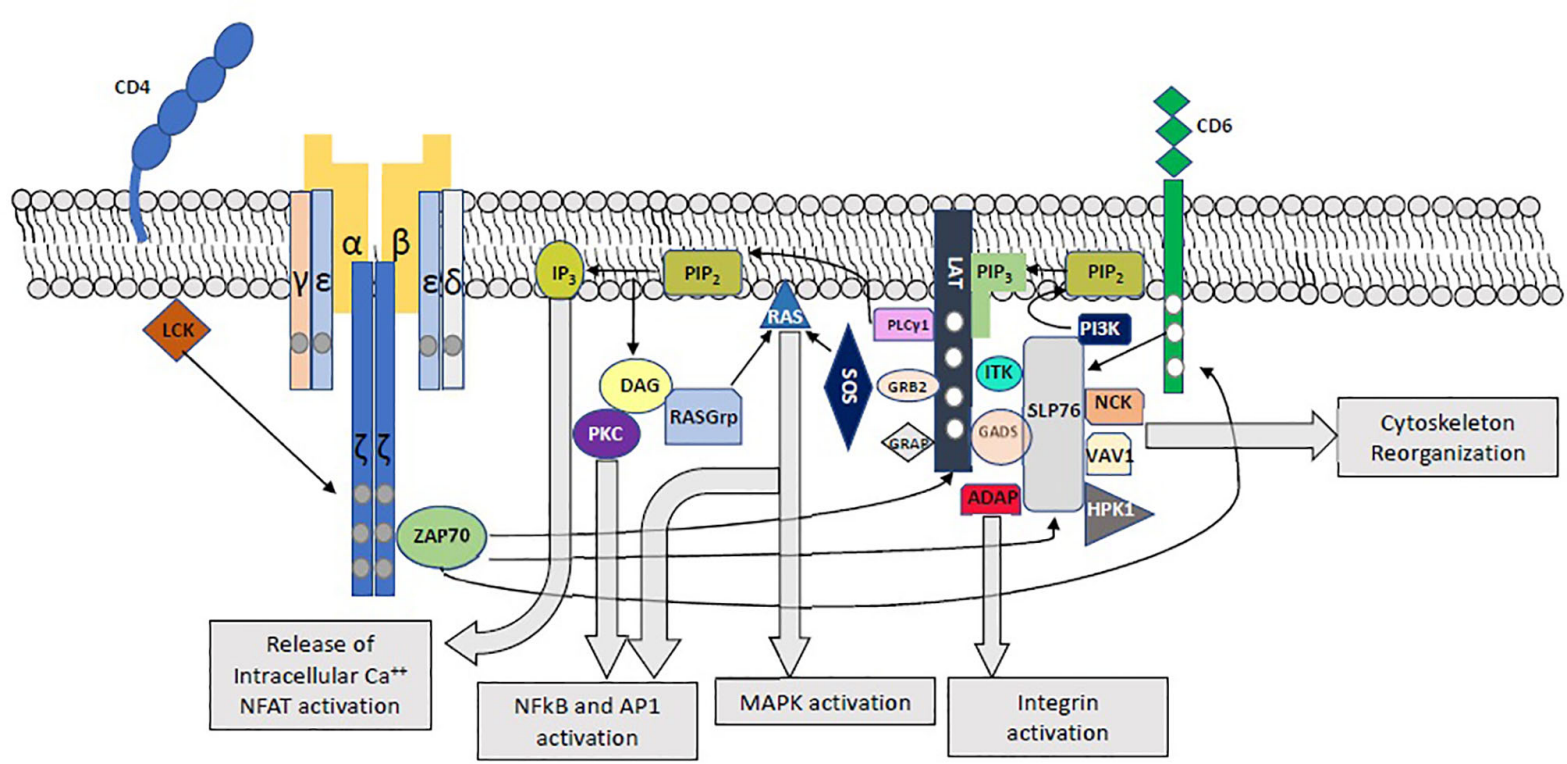
Proximal T-Cell Activation Signaling. The main proximal signaling events following engagement of the TCR are depicted, including the assembly of the LAT and SLP-76 signalosomes and their downstream effects.

Following engagement of the TCR, immunoreceptor tyrosine-based activation motifs (ITAMs) on CD3 cytoplasmic tails and ζ chains are phosphorylated by LCK, leading to the phosphorylation of ZAP70, which in turn phosphorylates LAT, SLP-76 and CD6 (1, 3–5) (**Figure 1**). Phosphorylation of the 4 distal tyrosine residues of LAT leads to the assembly of the LAT signalosome, including PLCγ1, Itk, SLP-76, Gads and Grb-2 (5–7). This signalosome mediates downstream events which are crucial for T-cell activation, including Calcium mobilization, Erk and NFAT activation, CD69 expression, and cytoskeletal organization (6).

Adaptor molecules lack both enzymatic and transcriptional activities. Through multiple interaction domains they function as modular scaffolds for the formation of multiprotein signaling complexes and have a vital role in transmitting and fine-tuning of T-cell activation. It is not surprising, therefore, to find human germline defects in these molecules that result in immune dysregulation.

Newly recognized inborn errors of immunity (IEI) due to monogenic mutations in several key adaptors in T-cell signaling have shed light on their function in humans. In this review, we summarize the current knowledge on adaptors in T-cell signaling for which human monogenic defects have been described, and discuss lessons learnt from comparison of cell lines, murine models and humans.

## Linker For Activation Of T Cells

LAT is expressed in T-, mast, NK- and immature B-cells, as well as megakaryocytes and platelets (6). This protein, with a short extracellular domain, a single transmembrane domain, and a long intracellular region, serves as a crucial nucleating factor for multiprotein signaling complexes. It plays a central role in T-cell activation downstream of the TCR, by recruiting kinases, effectors, and other adaptors into highly regulated signal transduction pathways. Upon TCR engagement, LAT is phosphorylated primarily by ZAP70, but also by Itk and Lck (6) in 4 conserved tyrosine residues (Y132, Y171 Y191 and Y226). Phosphorylated LAT binds to PLCγ1, SLP-76 (*via* Gads) and Grb-2, leading to the assembly of the LAT signalosomes (1, 5–7)(**Figure 1**). Phosphorylated tyrosine residues show predilection towards specific binding proteins. In this way, the Y132 residue binds PLCγ1 with greater affinity, while Grb2, Gads and Grap associate with the distal LAT phosphotyrosines Y171, Y191 and Y226 (6, 8–11). Nevertheless, each binding site specificity is not insulated, and the different SH2-containing LAT binding proteins show cooperative interactions: while Gads-SLP-76 binding stabilizes the LAT-PLCγ1 association and PLCγ1 activation through recruitment of Itk (12, 13), PLCγ1 stabilizes the binding of Grb2 to LAT. The cooperative interaction of LAT, Sos1 and Grb2, as well as LAT-Gads-SLP-76-ADAP allow for the assembly of macromolecular LAT signaling complexes, which are essential for T-cell signaling (6, 11, 14–16).

Once activated, PLCγ1 hydrolyzes phosphatidylinositol 4,5-bisphosphate (PIP_2_) to produce inositol 1,4,5-triphosphate (IP_3_) and diacylglycerol (DAG). DAG stimulates RasGrp, which in turn activates ERK1/2, and protein kinase C (PKC), leading to activation of the NFκB pathway. IP_3_ promotes the release of intracellular Ca^2+^ stores, leading to extracellular Ca^2+^ influx and NFAT activation. Grb2, through its constitutive association with Sos1 and Cbl, is involved in Ras and MAP kinase activation (6, 17). Moreover, by binding multiple Grb2 molecules to LAT, the Grb2-Sos1-LAT interaction mediates oligomerization of LAT. Importantly, LAT oligomerization was shown to be of greater significance for T-cell signaling under limiting stimulating conditions (14, 15).

SLP-76, another crucial adaptor, is recruited to the LAT signalosome *via* its constitutive interactions with Gads. In addition to its contribution to LAT- PLCγ1 interaction and PLCγ1 activation, this adaptor is involved in integrin and cytoskeletal function as well as downstream T-cell activation through multiple interactions with Nck, Vav1, Rac1, ADAP, Shb and p85 (5, 6, 17–19). Importantly, SLP-76 is also activated in a LAT-independent manner by phosphorylated CD6 (1, 20), and following integrin activation (21).

### LAT Murine Models

A complete LAT knockout murine model exhibited early arrest of thymocyte development in the double negative 3 (DN3) stage (6, 22). A LAT^4YF^ knock-in model, deficient in the 4 distal tyrosines showed a similar phenotype, indicating the importance of these 4 residues to pre-TCR signaling and T-cell development (23). In contrast, a point mutation in the Y136 residue (LAT^Y136F^, corresponding to human Y132 residue), impaired LAT binding to PLCγ1, Ca^2+^ flux and NFAT signaling, while the effect on ERK phosphorylation was variable (17, 24–27). Affected T-cells show severe, yet incomplete arrest in their development. Affected mice developed a polyclonal, Th2 lymphoproliferative disorder, with secondary massive expansion of eosinophils and B cells, multiorgan inflammation, autoimmune nephritis and fibrosis (24, 28–32). Aberrant T-cells exhibited an effector phenotype, with reduced proliferation in response to TCR stimulation, reduced FasL-mediated apoptosis, and a TCR^lo^CD5^hi^ phenotype, indicative of an abnormal survival and proliferation of autoreactive T-cells (24, 25, 30). In a knock-in murine model of the Y175, Y195 and Y235 residues (LAT^Y7/8/9F^ corresponding to the human Y171, Y191 and Y226 residues), a complete arrest of αβ-T cell alongside partial arrest of γδ T-cell development caused a similar albeit slightly delayed-onset lymphoproliferative Th2 disease (33). When the LAT^Y136F^ knock-in was crossed with TCRβ^-/-^ mice, a similar phenotype to the LAT^Y7/8/9F^ knock-in ensued, which demonstrates the importance of the LAT- PLCγ1 to both αβ and γδ T-cell maturation (29). Since recruitment of Grb2 and Gads is important for activation of PLCγ1, the difference between the LAT^Y136F^ and LAT^Y7/8/9F^ phenotypes can be attributed to differences in signal strength and the lower level of signaling required for γδ T-cell maturation (17).

While LAT^Y136F^ Tregs were non-functional, using adoptive transfer experiments with floxed genes, autoimmune phenotype in LAT mutated mice was proven to be intrinsic to effector CD4+ cells (27, 32) and independent of thymic development and of the distal 3 LAT tyrosine residues (1, 25, 27). Presumably, both the peripheral ablation of LAT in normally developed T-cells and the occurrence of peripheral LAT^Y136F^ T-cells enable a weak tonic TCR-dependent signal, leading to continued positive LAT-independent signaling events, including PI3K and SLP-76 activation, which is unopposed by a normal negative-feedback loop. This results in the emergence and expansion of an abnormal, autoreactive polyclonal Th2 subset (17, 25). Such negative feedback signals include Gab2-dependent SHP-2 activation and competitive inhibition of SLP-76 (34), Grap inhibition of ERK activation (35), SHIP-1-Dok1/2 mediated inhibition of Akt and Zap70 (36, 37), THEMIS-SHP1-Grb2 mediated regulation of proximal-TCR signaling, and LAT negative feedback of ZAP70 and CD3ζ phosphorylation (38), PTPN7 (38) and HPK-1 (39).

### LAT Inborn Errors of Immunity

Recently, human inborn errors of immunity (IEI) caused by monogenic defects in LAT have been described, with varying phenotypes: Bacchelli et al. (40) described 5 patients from a single consanguineous pedigree with severe combined immunodeficiency (SCID). All patients presented in early infancy with recurrent infections and failure to thrive (FTT), extremely low (<300 cells/mm3) T cell counts and absent T-cell proliferative response to phytohemagglutinin (PHA) and normal B and NK-cell counts. In one patient, increased γδ T-cell count was suspected. While all patients underwent hematopoietic stem cell transplantation (HSCT) using various donor types and conditioning regimens, 3/5 (60%) died of transplant related complications. A homozygous LAT c.44_45insT p.Leu16AlafsX28 mutation, associated with a complete loss of protein expression was found. Mutant LAT reconstitution in Jurkat cell lines was unable to restore post-stimulation CD69 expression, Ca^2+^ flux and downstream phosphorylation of SLP-76 and Vav1. TCR-induced apoptosis was severely reduced in LAT-deficient T-cell lines and was not restored following reconstitution with the LAT-mutant.

Keller et al. (41) described 3 siblings of a consanguineous family with a homozygous LAT c.268_269del mutation, which resulted in a premature stop-codon eliminating all major intracellular phosphorylation sites. While patients also presented in early infancy with recurrent infection including CMV viremia and recurrent pneumoniae resulting in bronchiectasis, their phenotype was notable for severe autoimmune cytopenias, anti-ADAMTS13^+^ microangiopathic hemolytic anemia, lymphoproliferation and an expansion of Th2-like effector T-cells, as well as elevated γδ T-cell counts, reminiscent of the partially deficient murine models (24, 33). Patients developed progressive hypogammaglobulinemia and CD4 and B cell lymphopenia with reductions in naïve CD4 and CD8 cells and reduced CD3 expression. TCR-dependent proliferation and activation was abrogated. While 2 patients died at 9 and 2 years of age of disseminated CMV infection and thrombotic thrombocytopenic purpura (TTP), respectively, one patient underwent a successful HSCT and is currently well.

Considering the elimination of all 4 major known phosphorylation sites, and the complete block in T-cell development in the equivalent murine model (23), it was somewhat surprising that Ca^2+^ mobilization and IκBα degradation, both downstream of PLCγ1 activation were normal in patients’ T cells, despite being affected in Jurkat cell lines (8, 41). While ERK phosphorylation was absent in patients’ CD4CD45Ro cells, ITK phosphorylation was normal both in patient’s cells and Jurkat-cell lines, in contrast to a previous report (42). The authors attribute the difference to the presence of a yet unknown LAT-replacing adaptor which is absent in Jurkat-cell lines. Indeed, the importance of LAT-independent TCR-signaling has been recognized previously (7, 20, 25, 38, 43–46). CD6 is known to recruit SLP-76, Gads, Grb2, Vav1 and SHIP1 independently of LAT (20, 47, 48), and is therefore a potential candidate for LAT-independent Itk phosphorylation and Calcium flux. While induced CD6 expression in Jurkat cell lines did not rescue Ca^2+^ mobilization in LAT-mutated cell-line, it is possible that this is because of lack of expression of the CD6 ligand (48). Possibly, CD6 or another LAT-substitute is responsible for Ca^2+^ mobilization downstream of PLCγ1, but is unable to replace ERK activation, which requires both PLCγ1 activation and an intact LAT-Grb2-Sos1 complex (6, 49). Another possible explanation lies in the partial rescue of LAT-dependent signaling *via* LCK binding to LAT upstream of the mutation site (50). Possibly, the impact of such binding is stronger in primary T-cells compared to Jurkat cell lines. Loss of RasGrp-induced ERK activation in turn, may have resulted in aberrant tonic T-cell signaling and basal TCRα levels in naïve CD4 cell (51), affecting LAT’s regulatory role in maintaining T-cell homeostasis. This is supported by the observation of low CD3 expression in LAT-mutated mice, Jurkat cell lines and human cells (41, 51, 52), as well as by the similar phenotype observed in RasGrp deficient patients (53). In this way, we postulate, that while LAT-independent signals are sufficient to allow for partial αβ and γδ T-cell development in the thymus, abnormally weak signal in the periphery results in aberrant tonic T-cell signaling and the development of a population of activated, dysregulated CD4+ cell.

The current concept for LAT function, is that LAT has both positive and negative roles in T-cell signaling: in the thymus, LAT is responsible for pre-TCR signaling and positive selection, and so complete loss of protein expression results in arrest of T-cell development. In the periphery, however, LAT has a dual role: on one hand it acts as a positive regulator of T-cell activation, including early T-cell activation, immune synapse development and cytoskeletal changes. On the other hand, LAT-dependent inhibition augments T-cell signaling and is involved in maintaining T-cell homeostasis (6, 51). Moreover, it is known that LAT functions as a central hub for the creation of multiple microclusters which then assemble into condensates. Recently, using affinity purification with mass spectrometry, LAT microclusters were proven to be heterogeneous. Many abortive or partially functional LAT signalosomes accompany the fully functional, high order signalosome. As such, it was shown, that 30 seconds after TCR engagement, LAT-SHIP1 containing signalosomes are much more abundant than LAT-SLP-76 signalosomes (47). Therefore, it is possible, that the different LAT signalosome isoforms have distinct functions, and that the net result of TCR-engagement depends on the combined output of these higher order LAT-signalosome-containing condensates. In this way, a partial deletion in LAT could potentially alter the composition of LAT-microclusters and attenuate the TCR signal.

## SH2 Domain-Containing Leukocyte Protein Of 76 KDa

SLP-76 is expressed in T cells, platelets, neutrophils, mast cells, macrophages and NK cells (54). It exerts its function through four distinct domains: an amino-terminal sterile α motif (SAM) domain is responsible for ACK1 binding and oligomerization (55, 56), followed by three tyrosine phosphorylation motifs, responsible for binding of multiple effectors, including Vav1, Nck, Itk and p85, thus promoting signal transduction and cytoskeletal organization (54). A central proline-rich domain includes the binding site for Gads, Grb2 and PLCγ1, and is responsible for the recruitment of PLCγ1 to the LAT signalosome and its activation by Itk (57). A C-terminal SH2 domain is involved in integrin function and the formation of LAT microclusters *via* ADAP (11, 16, 21, 58–60), as well as a negative-feedback loop through binding of HPK1 (11), and CD6 interaction (61).

An SLP-76 deficient Jurkat-cell line (denoted J14) demonstrated the importance of SLP-76 in PLCγ1 activation, intracellular Ca^2+^ flux, activation of the Ras, NFAT and AP1 pathways and early T-cell activation events, such as CD69 expression (62). Mutations in the three N-terminal tyrosine residues of SLP-76 (denoted SLP-76^Y3F^), as well as mutations of the Gads-binding site, all showed reduced Ca^2+^-dependent NFAT activation, ERK1/2 and PLCγ1 phosphorylation. A mutation in the SLP-76 SH2 domain, however, affected PLCγ1 phosphorylation to the same extent, while NFAT activation was variably affected and ERK phosphorylation was comparable to wild type (60, 63).

While there is a 60% perinatal mortality rate among SLP-76 deficient mice, the remaining suffer from defects in T-cell development, as well as mast cell, neutrophil, platelet and vascular defects (54, 64–67). SLP-76 deficient T-cells show arrested thymic development at the DN3 stage, in a similar manner to LAT-deficient mice. An SLP-76-N-Terminal domain depleted murine model showed a similar phenotype to the SLP-76^-/-^ model (68). On the other hand, knock-in mutations in the N-terminal and proline-rich domains, including the SLP-76^Y3F^, SLP-76^Y112/128F^, SLP-76^Y145F^ knock-ins, and selective deletions of the Gads-binding site all resulted in varying degrees of aberrant, yet not obliterated thymic differentiation, impaired calcium flux, actin polymerization and PLCγ1 and ERK phosphorylation (58, 68, 69). Deletion of the SH2 domain results in a milder impairment in thymocyte development, near normal Ca^2+^ flux and ERK activation, but defective T-cell proliferation and activation, reminiscent of the ADAP-deficient murine model (19, 68, 69).

### SLP-76 Inborn Errors of Immunity

Recently, a single Palestinian patient was described with a novel homozygous mutation c.957+1G>A; p.K309FSx17 in SLP-76, affecting a donor splice site and resulting in skipping of exon 14 and deletion of the C-terminal domain (70). Clinically, the patient presented in early infancy with a combination of autoimmune and lymphoproliferative manifestations, CMV viremia, skin disease, Aspergillus fumigatus brain abscesses and local BCGitis. His immune phenotype included a skewed CD4:CD8 ratio, clonal expansion of central memory CD4+ cells and terminally differentiated CD8+ cells, and a skewed T-cell repertoire, alongside a severe neutrophil defect, an NK functional defect and arrest of B-cell development. A defect in platelet aggregation led to a petechial rash. The patient underwent a haplo-identical HSCT at the age of 10 months. However, he died in the immediate post-HSCT period of transplant related complications.

While there was no protein expression in patient’s peripheral blood mononuclear cells (PBMCs), there was somewhat lower expression of the SLP-76 in J14 reconstituted with the mutant protein. The differential expression between patient cells and reconstituted Jurkat cell lines could be attributed to fixed promoter-driven cDNA expression in Jurkat cell lines, as well as lower expression of SLP-76 in patient’s clonally expanded, terminally differentiated cells. The authors concluded that the mutation results in a hypomorphic, unstable yet partially functional protein, leading to reduced TCR-dependent ERK, S6 and PLCγ1 phosphorylation, abnormal Ca^2+^ flux and poor upregulation of CD69, CD25 and CD98. This hypothesis is supported by the hypomorphic phenotype associated with SH2-domain defects (54, 63). Partial T-cell developmental arrest and immune dysregulation were also noted in a murine model of ~90% reduced SLP-76 levels (71), pointing to the possible association between reduced SLP-76 signaling and immune dysregulation. Interestingly, in contrast to the murine phenotype, B-cell arrest was evident in the patient. SLP-76 is known to be involved in pre-B cell signaling (72). Other defects in pre-BCR signaling also show a discrepancy between the mouse and human phenotype, perhaps pointing to the more stringent requirements for human pre-BCR signaling as compared to mice (73).

## Adhesion And Degranulation Adaptor Protein

ADAP, also termed FYB and SLP130, is expressed in T-cells and myeloid cells. This protein contains a proline-rich region and an SH3-like domain, both of which bind SKAP55. A phosphotyrosine motifs rich domain is responsible for binding FYN and SLP-76, and an Ena/VASP-homology1 (EVH1)-binding domain binds Ena/VASP family proteins (74). Upon T-cell activation, ADAP is phosphorylated at 3 tyrosine residues by FYN, followed by binding to SLP-76 through its SH2 domain (6, 59, 74). Through its association with SLP-76, ADAP contributes to the cross-linking of LAT molecules into microclusters and amplification of proximal signaling events (11, 16). This protein has also been implicated in regulation of the assembly of the CBM (CARMA1/Bcl10/MALT1) complex, leading to NFkB activation (19), as well as integrin activation (19, 21, 74, 75). In CD8+ cells, ADAP was associated with increased PD1 expression and reduced anti-tumor immunity (76), pointing to a possible regulatory role (77). ADAP^-/-^ mice show moderate thrombocytopenia and mildly decreased thymocyte numbers (78). Despite normal proximal TCR-signaling events, ADAP-/- T cells have abnormal activation and proliferation and impaired LFA-1 clustering in response to TCR stimulation. It is somewhat surprising then, that ADAP deficiency in humans, which is associated with congenital autosomal-recessive small-platelet thrombocytopenia (CARST) (79–82), has no known immune defect. Activation-dependent, raft recruited ADAP-like phosphoprotein (ARAP) shares sequence homology with ADAP and activates integrin in a TCR- and SLP-76-dependent manner (83). Therefore, it is a possible candidate for rescuing ADAP function in T-cells.

## Conclusion

The function of adaptor molecules in T-cell signaling has been thoroughly investigated. However, recent discoveries of human inborn errors of immunity have raised further questions regarding the differential function and compensatory mechanisms in T-cell signaling complexes between murine models, Jurkat cells and patients. Further research is needed to answer these questions.

## Author Contributions

YD-S wrote the paper. PS initiated the idea for review and reviewed the paper. IZ reviewed the paper. HM-S reviewed the paper. All authors contributed to the article and approved the submitted version.

## Conflict of Interest

The authors declare that the research was conducted in the absence of any commercial or financial relationships that could be construed as a potential conflict of interest.

## Publisher’s Note

All claims expressed in this article are solely those of the authors and do not necessarily represent those of their affiliated organizations, or those of the publisher, the editors and the reviewers. Any product that may be evaluated in this article, or claim that may be made by its manufacturer, is not guaranteed or endorsed by the publisher.
